# Granulocyte colony-stimulating factor-induced isolated hyperbilirubinemia in acute myelogenous leukemia: A case report

**DOI:** 10.1097/MD.0000000000044637

**Published:** 2025-09-19

**Authors:** Jawad K. Zrein, Doha A. Houcheimy, Ahmad S. Khalil

**Affiliations:** a Department of Hematology-Oncology, Faculty of Medicine and Medical Sciences, University of Balamand, Beirut, Lebanon; b Division of Hematology and Oncology, Mount Lebanon Hospital University Medical Center, Beirut, Lebanon; c Faculty of Medicine and Medical Sciences, University of Balamand, Beirut, Lebanon.

**Keywords:** acute myelogenous leukemia, granulocyte colony-stimulating factor, hyperbilirubinemia

## Abstract

**Rationale:**

Granulocyte colony-stimulating factors (G-CSFs) are used in treating chemotherapy-induced neutropenia or in healthy stem cell donors before collection since they mobilize hematopoietic stem cells from the bone marrow into the peripheral blood. Reported side effects of these drugs include bone pain, fatigue, and, rarely, splenic rupture. However, G-CSFs were not previously associated with isolated hyperbilirubinemia.

**Patient concerns:**

We describe the case of a previously healthy 42-year-old female who presented to the emergency department with fever and chills. Initial laboratory investigations revealed pancytopenia along with normal liver, renal, and bilirubin levels.

**Diagnoses:**

A peripheral smear showed an elevated blast cell count, and subsequent bone marrow cytology and flow cytometry confirmed the diagnosis of acute myelogenous leukemia.

**Interventions:**

The patient was started on induction chemotherapy and subsequently developed febrile neutropenia, for which G-CSF was initiated to promote neutrophil recovery.

**Outcomes:**

Following G-CSF initiation, the patient demonstrated a progressive rise in total bilirubin, while liver enzymes (alanine aminotransferase, aspartate aminotransferase, γ-glutamyl transpeptidase) and other markers of liver function remained within normal limits. Extensive evaluation for alternative causes of hyperbilirubinemia was unremarkable, so given the temporal association and lack of other identifiable etiologies, G-CSF-induced isolated hyperbilirubinemia was suspected.

**Lessons:**

This case highlights a potential and previously unreported adverse effect of G-CSF. Clinicians should be aware of this possible association, particularly in patients undergoing treatment for hematologic malignancies.

**Key points**

- Recognition of G-CSF as a rare cause of hyperbilirubinemia.
- Proposed mechanisms of G-CSF induced hyperbilirubinemia.
- Clinical implications and management considerations.

## 1. Introduction

Granulocyte colony-stimulating factors (G-CSFs) are glycoproteins that stimulate the bone marrow to produce neutrophils. They are clinically effective in treating neutropenia, especially in cancer patients undergoing cytotoxic chemotherapy or bone marrow transplantation, and have been shown to mobilize hematopoietic stem cells into the blood for transplantation. In addition, G-CSFs are used for supportive care in hematological malignancies.^[1–3]^ Furthermore, G-CSFs also enhance neutrophil functions such as phagocytosis and microbial clearance.^[1,2]^

Although G-CSFs are generally safe, they may cause side effects when administered. Reported side effects include bone pain and mild fatigue.^[2]^ Rare adverse effects include splenic rupture, pulmonary toxicity, capillary leak syndrome, and thrombocytopenia.^[2,4]^ While hepatic dysfunction, most notably hyperbilirubinemia, is an unusual and rare effect of these drugs in the literature, its recognition may be clinically useful. This report describes a case of G-CSF-associated hyperbilirubinemia, aiming to clarify this rare complication and its relevance in the field.

## 2. Case presentation

A 42-year-old female with no significant past medical history presented for fever and chills. Initial laboratory evaluation showed pancytopenia. A peripheral smear demonstrated an elevated blast count, suggesting an underlying bone marrow process abnormality. Liver function tests, bilirubin level, renal function tests, and electrolytes were within normal limits at presentation. A diagnosis of acute myelogenous leukemia was confirmed by bone marrow cytology and flow cytometry.

On day 6 of hospitalization, the patient developed new-onset right upper quadrant abdominal pain and recurrent fevers. Abdominal ultrasound revealed findings consistent with acute calculous cholecystitis, including a distended gallbladder with multiple gallstones, a thickened gallbladder wall, and no evidence of biliary obstruction. At that time, her liver function tests showed mild transaminitis (aspartate aminotransferase [AST] 161 U/L and alanine aminotransferase [ALT] 102 U/L) with normal alkaline phosphatase (ALP 95 U/L) and γ-glutamyl transpeptidase 144 U/L. The total bilirubin level was 0.58 mg/dL and direct bilirubin was 0.28 mg/dL. Given her pancytopenia and poor surgical candidacy, a percutaneous cholecystostomy tube was placed, leading to clinical stabilization.

On hospital day 12, standard induction chemotherapy was started (7 + 3 regimen, consisting of cytarabine administered continuously for 7 days and daunorubicin given the first 3 days). By this time, her liver function tests had largely returned to baseline: total bilirubin 0.86 mg/dL, direct bilirubin 0.45 mg/dL, AST 36 U/L, ALT 53 U/L, and ALP 105 U/L with only γ-glutamyl transpeptidase elevated at 237 U/L. The patient initially tolerated chemotherapy well. On day 10 of chemotherapy (hospital day 22), G-CSF was started at a dose of 300 μg daily to support her white blood cell recovery, as her WBC count had dropped to 0.08 × 10^3^/μL (normal: 4 × 10^3^/μL).

The patient’s last bilirubin levels 2 days before G-CSF administration (hospital day 19) were 1.34 mg/dL (total), and 0.55 mg/dL (direct), and all liver function tests within normal limits (AST 7 U/L, ALT 13 U/L, ALP 67 U/L), reducing the likelihood that chemotherapy was responsible for the subsequent hyperbilirubinemia.^[5]^ By the second day of G-CSF therapy (hospital day 23), the patient’s total bilirubin had begun to rise, reaching 3.08 mg/dL, with a direct bilirubin of 1.73 mg/dL. Clinically, she developed noticeable jaundice, scleral icterus, dark urine, and pruritus.

**Figure 1.**
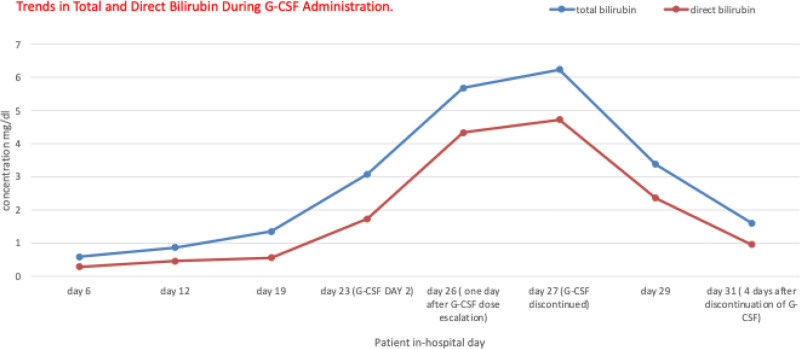
Trends in total and direct bilirubin during G-CSF administration. Bilirubin levels began to rise shortly after G-CSF initiation and decrease after discontinuation.

Despite these concerning findings, 3 days later (hospital day 25), her G-CSF dose was increased to 300 μg twice daily; this decision was primarily based on the persistently low neutrophil count (WBC count was 0.05 × 10^3^/μL), but also because G-CSF had not yet been suspected as the cause of her isolated hyperbilirubinemia. This dose escalation coincided with a more dramatic rise in her bilirubin levels the following day (hospital day 26), peaking at 5.68 mg/dL (total) and 4.33 mg/dL (direct) (see Fig. 1).

A repeat abdominal ultrasound was performed to evaluate for potential hepatic or biliary obstruction. Imaging showed no evidence of intrahepatic or extrahepatic biliary duct dilation. The liver appeared normal in size and texture, and the gallbladder was collapsed over the cholecystostomy tube without additional abnormalities. With no identifiable hepatobiliary obstruction or other hepatic dysfunction, the pattern and timing of bilirubin elevation raised suspicion for G-CSF-induced hyperbilirubinemia.

As the patient’s bilirubin levels continued to rise, reaching a peak of 6.24 mg/dL (total) and 4.73 mg/dL (direct), G-CSF therapy was discontinued at day 6 (hospital day 27). Remarkably, her bilirubin levels began to decrease within 2 days, reaching 3.38 mg/dL (total) and 2.36 mg/dL (direct). Four days after discontinuation, her bilirubin levels returned to near-normal levels of 1.6 mg/dL (total) and 0.95 mg/dL (direct) (see Fig. 1). Following that, the patient received 4 cycles of cytarabine, during which no G-CSF support was required (WBC count was 5.2 × 10^3^/μL). The patient is now in complete remission from acute myelogenous leukemia.

## 3. Discussion

Drug-induced hyperbilirubinemia has been reported with several drugs such as statins, protease inhibitors, and many others via a reduced hepatic uptake of bilirubin or impaired bilirubin conjugation.^[6,7]^ Moreover, certain medications, such as bile salt export pumps, have been described to cause cholestasis and direct hyperbilirubinemia.^[6]^ Direct hepatocyte injury, as seen with acetaminophen toxicity, can also lead to bilirubin release into the bloodstream due to hepatocyte lysis and release of intracellular contents.^[6,7]^ However, to our knowledge, G-CSF-induced hyperbilirubinemia has not been previously reported in the literature.

The sparsity of similarly reported incidences of this rare adverse effect and the lack of literature on suggestive mechanisms make it hard to establish a definitive causal relationship between G-CSF and hyperbilirubinemia. Nevertheless, several preclinical studies have explored G-CSF effects on liver function and hepatic regeneration which may suggest a potential mechanism for this adverse effect. In a study on patients with acute-on-chronic liver failure, it was shown that G-CSF therapy improved liver function, demonstrated by reduced Child-Turcotte-Pugh, Model for End-Stage Liver Disease and Sequential Organ Failure Assessment scores.^[6]^ In animal models with liver damage, G-CSF was found to facilitate hematopoietic stem cell mobilization from the bone marrow and their migration to the liver, where they may differentiate into hepatocyte-like or supporting cell types. In addition, there is evidence of bone marrow derived cells integrating into liver tissue and contributing to hepatic repair and regeneration through oval cells and CD34+ cells in the same animal models.^[6]^

Additionally, experimental models show that G-CSF promotes both hepatocyte proliferation and activation of hepatic progenitor cells through the upregulation of gene expression of regenerative markers such as hepatocyte growth factor and Nanog homeobox.^[8]^ These regenerative pathways have primarily been described in murine models and are not yet clearly demonstrated in humans.

On the other hand, conflicting evidence is also present refuting a direct association between the G-CSF and hyperbilirubinemia. A study by Ortega et al demonstrated no significant improvement in clinical outcomes or hepatic function with G-CSF administration. G-CSF treatment was associated with an increase in extracellular matrix remodeling, exacerbated inflammatory responses, inflammasome activation, neutrophil infiltration, and oxidative stress surge in the liver, implying both beneficial and detrimental outcomes.^[8]^ However, some of these responses may be triggering a cellular response that alters the structural components of liver tissue, causing excessive inflammation and tissue damage that eventually leads to liver injury.^[8]^ This shows the inconsistency of the G-CSF regenerative effect.

## 4. Conclusion

In our case, the temporal relationship between G-CSF administration and the progressive rise in isolated bilirubin levels, followed by prompt improvement after drug discontinuation, strongly supports a potential adverse effect of G-CSF. While the underlying mechanism remains speculative, this case underscores the need for clinician awareness of a possible hepatic side effect, particularly when encountering unexplained hyperbilirubinemia in patients receiving G-CSF, and the potential risk when prescribing G-CSF in patients with preexisting liver conditions.

## Author contributions

**Writing – original draft:** Jawad K. Zrein, Doha A. Houcheimy.

**Writing – review & editing:** Ahmad S. Khalil.

## References

[1] Anderlini P Champlin RE. Biologic and molecular effects of granulocyte colony-stimulating factor in healthy individuals: recent findings and current challenges. Blood. 2008;111:1767–72. 18057230 10.1182/blood-2007-07-097543

[2] Root RK Dale DC. Granulocyte colony-stimulating factor and granulocyte‐macrophage colony-stimulating factor: comparisons and potential for use in the treatment of infections in nonneutropenic patients. J Infect Dis. 1999;179:S342–52. 10081506 10.1086/513857

[3] Dale DC Crawford J Klippel Z. A systematic literature review of the efficacy, effectiveness, and safety of filgrastim. Support Care Cancer. 2018;26:7–20. 28939926 10.1007/s00520-017-3854-x PMC5827957

[4] Kango G Haroun F. Filgrastim induced thrombocytopenia. BMJ Case Rep. 2020;13:e234584. 10.1136/bcr-2020-234584 PMC7328747 32606115

[5] Feng A Masadeh M Murali AR. Recurrent isolated hyperbilirubinemia from drug-induced impaired hepatic bilirubin uptake. Hepatology. 2020;72:1145–7. 32003900 10.1002/hep.31154

[6] Chavez-Tapia NC Mendiola-Pastrana I Ornelas-Arroyo VJ. Granulocyte-colony stimulating factor for acute-on-chronic liver failure: systematic review and meta-analysis. Ann Hepatol. 2015;14:631–41. 26256891

[7] Tátrai P Krajcsi P. Prediction of drug-induced hyperbilirubinemia by in vitro testing. Pharmaceutics. 2020;12:755. 32796590 10.3390/pharmaceutics12080755 PMC7465333

[8] Ortega-Ribera M Zhuang Y Brezani V. G-CSF increases calprotectin expression, liver damage and neuroinflammation in a murine model of alcohol-induced ACLF. Front Cell Dev Biol. 2024;12:1347395. 38419842 10.3389/fcell.2024.1347395 PMC10899467

